# *catena*-Poly[[[aqua­(pyrazino­[2,3-*f*][1,10]phenanthroline-κ^2^ *N* ^8^,*N* ^9^)zinc(II)]-μ-penta­nedioato] monohydrate]

**DOI:** 10.1107/S1600536810042340

**Published:** 2010-11-06

**Authors:** Wei Fang

**Affiliations:** a Department of Chemistry, Baicheng Normal University, Baicheng 137000, People’s Republic of China

## Abstract

In the title compound, {[Zn(C_5_H_6_O_4_)(C_14_H_8_N_4_)(H_2_O)]·H_2_O}_*n*_, the Zn^2+^ ion is coordinated by an *N*,*N*′-bidentate pyrazino­[2,3-*f*][1,10]phenanthroline (pyphen) ligand, a water molecule and a monodentate glutarate (glu) dianion. A symmetry-generated *O*:*O*′-bidentate glu dianion completes a distorted *cis*-ZnN_2_O_4_ octa­hedral coordination geometry for the metal ion. The bridging glu species generates [110] polymeric chains in the crystal. O—H⋯O hydrogen bonds involving both the coordinated and uncoordinated water mol­ecules help to consolidate the structure and neighbouring pyphen units inter­act through numerous aromatic π–π inter­actions [minimum centroid–centroid separation = 3.654 (3) Å], resulting in a two-dimensional network.

## Related literature

For the synthesis of the ligand, see: Dickeson & Summers (1970 ▶). For related structures, see: Fang-Wei & Mei (2007 ▶); Li *et al.* (2006 ▶).
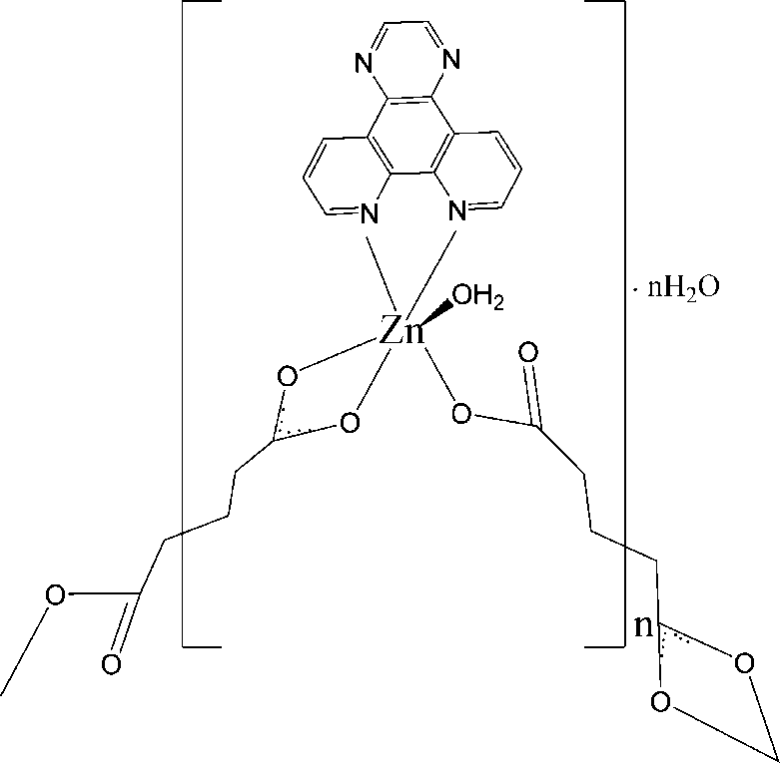


## Experimental

### 

#### Crystal data

[Zn(C_5_H_6_O_4_)(C_14_H_8_N_4_)(H_2_O)]·H_2_O
*M* *_r_* = 463.74
Triclinic, 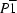

*a* = 6.397 (3) Å
*b* = 9.384 (5) Å
*c* = 16.409 (8) Å
α = 98.067 (5)°
β = 100.859 (5)°
γ = 101.274 (5)°
*V* = 932.5 (8) Å^3^
*Z* = 2
Mo *K*α radiation
μ = 1.37 mm^−1^
*T* = 292 K
0.78 × 0.52 × 0.36 mm

#### Data collection

Bruker SMART CCD diffractometer
Absorption correction: multi-scan (*SADABS*; Bruker, 2002 ▶) *T*_min_ = 0.432, *T*_max_ = 0.611
8019 measured reflections
3702 independent reflections
2929 reflections with *I* > 2σ(*I*)
*R*_int_ = 0.062

#### Refinement

*R*[*F* ^2^ > 2σ(*F* ^2^)] = 0.040
*wR*(*F* ^2^) = 0.100
*S* = 0.98
3702 reflections
283 parameters
H atoms treated by a mixture of independent and constrained refinement
Δρ_max_ = 0.86 e Å^−3^
Δρ_min_ = −0.59 e Å^−3^

### 

Data collection: *SMART* (Bruker, 2002 ▶); cell refinement: *SAINT* (Bruker, 2002 ▶); data reduction: *SAINT*; program(s) used to solve structure: *SHELXS97* (Sheldrick, 2008 ▶); program(s) used to refine structure: *SHELXL97* (Sheldrick, 2008 ▶); molecular graphics: *SHELXTL* (Sheldrick, 2008 ▶); software used to prepare material for publication: *SHELXTL*.

## Supplementary Material

Crystal structure: contains datablocks global, I. DOI: 10.1107/S1600536810042340/hb5645sup1.cif

Structure factors: contains datablocks I. DOI: 10.1107/S1600536810042340/hb5645Isup2.hkl

Additional supplementary materials:  crystallographic information; 3D view; checkCIF report

## Figures and Tables

**Table 1:** Selected bond lengths (Å)

Zn1—O2	1.987 (2)
Zn1—N1	2.120 (3)
Zn1—O5	2.137 (2)
Zn1—O4^i^	2.154 (2)
Zn1—N2	2.188 (2)
Zn1—O3^i^	2.347 (2)

Symmetry code: (i) 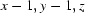.

**Table 2:** Hydrogen-bond geometry (Å, °)

*D*—H⋯*A*	*D*—H	H⋯*A*	*D*⋯*A*	*D*—H⋯*A*
O5—H5*A*⋯O*W*1^ii^	0.82	1.89	2.702 (3)	173
O5—H5*B*⋯O4^iii^	1.00 (4)	1.91 (4)	2.856 (3)	157 (3)
O*W*1—H*WA*1⋯O4^iv^	0.85 (4)	2.03 (4)	2.840 (4)	161 (3)
O*W*1—H*WBA*⋯O2	0.79 (4)	1.95 (4)	2.733 (4)	170 (4)

Symmetry codes: (ii) 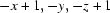; (iii) 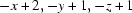; (iv) 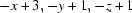.

**Fig. 1.**
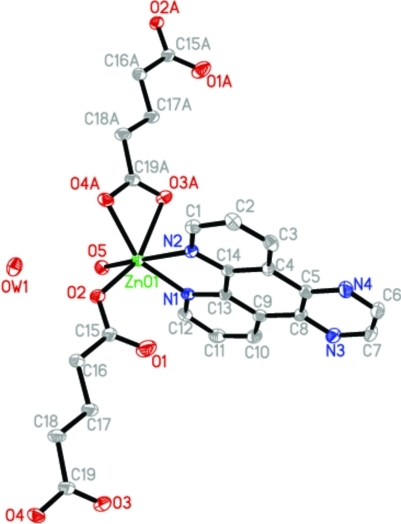
view of the local coordination of Zn(II) with displacement ellipsoids drawn at the 30% probability level.

**Fig. 2.**
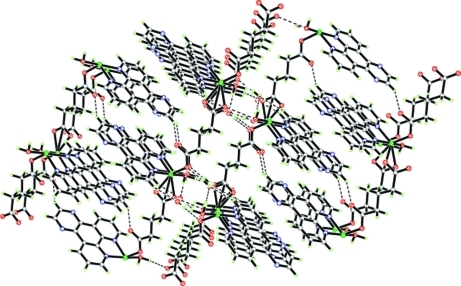
A view of the two-dimensional supramolecular structure of (I) generated by π-π interactions and hydrogen-bonding.

## supplementary crystallographic information

### Comment

The 1,10-phenanthroline (phen) ligand and its derivatives are important ligands with numerous uses in the construction of metal-organic complexes.Supramolecular architectures baswd on the phen derivative pyrazino[2,3-*f*][1,10]phenanthroline(PyPhen) molecule have considerably less attention (Li *et al.*,2006) A s part of our ongoing studies in this area (Fang-Wei & Mei, 2007). We selected glutaric acid (C_5_H_6_O_4_^2-^) to act as a metal-metal linker in its deprotonated form and *L* as a secondary ligand, generating the title compound, [Zn(C_14_H_8_N_4_)(C_5_H_6_O_4_)(H_2_O).H_2_O], a new coordinationg polymer, which is reported here. In compound (I), the Zn^II^ atom of unit is surrounded by two N atoms derived from the bidentate PyPhen ligand, three O atoms from two glutaric acid dianions (one monodentate, one bidentate) and one water molecule (Figure 1, Table 1) a distorted octahedral *cis*-ZnN_2_O_4_arrangement is formed. Neighboring Zn^II^ atoms are bridged by the centrosymmetric glutaric acid ligands forming a one-dimensional chain structure (Fig.2). In the crystal structure, adjacent chains are connected through π-π interactions between PyPhen and PyPhen ligands with a minimum centroid -centroid stacking distance of 3.372 Å. O—H···O hydrogen bonds involving the water molecules and carboxylate O atom acceptors (Table 2) complete the structure.

### Experimental

The pyphen ligand was synthesized according to the literature method of Dickeson & Summers (1970). A mixture of ZnCl_2_ (0.3 mmol), pyphen (0.1 mmol) and glutaric acid (0.3 mmol) in distilled water (30 ml) was stirred thoroughly for 1 h at ambient temperature. The pH was adjusted to 7.5 with aqueous NaOH solution. The suspension was then sealed in a Teflon-lined stainless steel reaction vessel (40 ml). The reaction was performed under autogeneous pressure and static conditions in an oven at 443 K for 4.5 d. The vessel was then cooled slowly inside the oven to 298 K at a rate of 5 K h^-1^ before opening: amaranth (red) blocks of (I) were collected.

### Refinement

All H atoms on C atoms were generated geometrically and refined as riding atoms with C—H= 0.93Å and *U*_iso_(H)= 1.2 times *U*_eq_(C).

### Crystal data

**Table:**

[Zn(C_5_H_6_O_4_)(C_14_H_8_N_4_)(H_2_O)]·H_2_O	*V* = 932.5 (8) Å^3^
*M**_r_* = 463.74	*Z* = 2
Triclinic, *P*1	*F*(000) = 476
Hall symbol: -P 1	*D*_x_ = 1.652 Mg m^−^^3^
*a* = 6.397 (3) Å	Mo *K*α radiation, λ = 0.71073 Å
*b* = 9.384 (5) Å	θ = 2.0–26.3°
*c* = 16.409 (8) Å	µ = 1.37 mm^−^^1^
α = 98.067 (5)°	*T* = 292 K
β = 100.859 (5)°	Block, amaranth
γ = 101.274 (5)°	0.78 × 0.52 × 0.36 mm

### Data collection

**Table:**

Bruker SMART CCD diffractometer	3702 independent reflections
Radiation source: fine-focus sealed tube	2929 reflections with *I* > 2σ(*I*)
graphite	*R*_int_ = 0.062
Detector resolution: 0 pixels mm^-1^	θ_max_ = 26.1°, θ_min_ = 2.3°
ω scans	*h* = −7→7
Absorption correction: multi-scan (*SADABS*; Bruker, 2002)	*k* = −11→11
*T*_min_ = 0.432, *T*_max_ = 0.611	*l* = −20→20
8019 measured reflections	

### Refinement

**Table:**

Refinement on *F*^2^	Primary atom site location: structure-invariant direct methods
Least-squares matrix: full	Secondary atom site location: difference Fourier map
*R*[*F*^2^ > 2σ(*F*^2^)] = 0.040	Hydrogen site location: constr
*wR*(*F*^2^) = 0.100	H atoms treated by a mixture of independent and constrained refinement
*S* = 0.98	*w* = 1/[σ^2^(*F*_o_^2^) + (0.0464*P*)^2^] where *P* = (*F*_o_^2^ + 2*F*_c_^2^)/3
3702 reflections	(Δ/σ)_max_ = 0.001
283 parameters	Δρ_max_ = 0.86 e Å^−^^3^
0 restraints	Δρ_min_ = −0.59 e Å^−^^3^

### Special details

**Table:**

Geometry. All e.s.d.'s (except the e.s.d. in the dihedral angle between two l.s. planes) are estimated using the full covariance matrix. The cell e.s.d.'s are taken into account individually in the estimation of e.s.d.'s in distances, angles and torsion angles; correlations between e.s.d.'s in cell parameters are only used when they are defined by crystal symmetry. An approximate (isotropic) treatment of cell e.s.d.'s is used for estimating e.s.d.'s involving l.s. planes.
Refinement. Refinement of *F*^2^ against ALL reflections. The weighted *R*-factor *wR* and goodness of fit *S* are based on *F*^2^, conventional *R*-factors *R* are based on *F*, with *F* set to zero for negative *F*^2^. The threshold expression of *F*^2^ > σ(*F*^2^) is used only for calculating *R*-factors(gt) *etc*. and is not relevant to the choice of reflections for refinement. *R*-factors based on *F*^2^ are statistically about twice as large as those based on *F*, and *R*- factors based on ALL data will be even larger.

### Fractional atomic coordinates and isotropic or equivalent isotropic displacement parameters (Å^2^)

**Table:**

	*x*	*y*	*z*	*U*_iso_*/*U*_eq_	
Zn1	0.71632 (5)	0.09770 (4)	0.66833 (2)	0.02738 (13)	
O5	0.4196 (3)	0.1252 (2)	0.59395 (14)	0.0354 (5)	
H5A	0.3260	0.0479	0.5847	0.053*	
O3	1.5203 (3)	0.8764 (2)	0.69454 (14)	0.0384 (5)	
O4	1.6651 (3)	0.8894 (2)	0.58459 (13)	0.0367 (5)	
OW1	0.9119 (4)	0.1180 (3)	0.44343 (17)	0.0424 (6)	
N2	0.9708 (4)	0.0659 (3)	0.76912 (15)	0.0270 (5)	
N1	0.6579 (4)	0.2190 (3)	0.77760 (15)	0.0271 (5)	
N3	0.9142 (5)	0.3624 (3)	1.07957 (17)	0.0425 (7)	
C4	1.1058 (5)	0.1251 (3)	0.92035 (18)	0.0290 (7)	
N4	1.2336 (5)	0.1937 (3)	1.07170 (17)	0.0410 (7)	
C14	0.9630 (4)	0.1335 (3)	0.84604 (18)	0.0249 (6)	
C8	0.9326 (5)	0.2869 (3)	1.00470 (19)	0.0325 (7)	
C13	0.7948 (5)	0.2178 (3)	0.85096 (18)	0.0259 (6)	
C12	0.5054 (5)	0.2965 (3)	0.7799 (2)	0.0340 (7)	
H12A	0.4108	0.2974	0.7295	0.041*	
C9	0.7811 (5)	0.2942 (3)	0.92828 (19)	0.0303 (7)	
C5	1.0920 (5)	0.2042 (3)	1.00158 (18)	0.0327 (7)	
C16	1.1651 (5)	0.4264 (3)	0.59565 (19)	0.0343 (7)	
H16A	1.0786	0.4284	0.5407	0.041*	
H16B	1.2742	0.3709	0.5865	0.041*	
C19	1.5487 (5)	0.8164 (3)	0.6267 (2)	0.0323 (7)	
C15	1.0173 (5)	0.3451 (3)	0.6441 (2)	0.0352 (7)	
C10	0.6172 (5)	0.3738 (3)	0.9281 (2)	0.0383 (8)	
H10A	0.6016	0.4249	0.9786	0.046*	
C2	1.2673 (5)	−0.0301 (4)	0.8352 (2)	0.0376 (8)	
H2A	1.3682	−0.0879	0.8294	0.045*	
C17	1.2817 (6)	0.5833 (3)	0.6374 (2)	0.0400 (8)	
H17A	1.3581	0.5832	0.6944	0.048*	
H17B	1.1738	0.6422	0.6414	0.048*	
C3	1.2606 (5)	0.0399 (3)	0.9126 (2)	0.0350 (7)	
H3B	1.3586	0.0314	0.9604	0.042*	
C11	0.4807 (5)	0.3765 (4)	0.8541 (2)	0.0408 (8)	
H11A	0.3733	0.4306	0.8532	0.049*	
C18	1.4442 (5)	0.6551 (3)	0.5904 (2)	0.0391 (8)	
H18A	1.5587	0.6008	0.5907	0.047*	
H18B	1.3701	0.6468	0.5321	0.047*	
C1	1.1200 (5)	−0.0139 (3)	0.7642 (2)	0.0331 (7)	
H1A	1.1268	−0.0610	0.7113	0.040*	
C7	1.0537 (6)	0.3505 (4)	1.1468 (2)	0.0487 (9)	
H7A	1.0475	0.3999	1.1992	0.058*	
C6	1.2114 (6)	0.2665 (4)	1.1429 (2)	0.0490 (10)	
H6A	1.3045	0.2621	1.1929	0.059*	
O2	0.8998 (3)	0.2189 (2)	0.60661 (13)	0.0366 (5)	
O1	1.0269 (5)	0.3952 (3)	0.71797 (17)	0.0684 (9)	
HWBA	0.923 (6)	0.152 (4)	0.492 (2)	0.044 (12)*	
H5B	0.432 (6)	0.129 (4)	0.534 (2)	0.068 (12)*	
HWA1	1.048 (6)	0.128 (4)	0.446 (2)	0.047 (11)*	

### Atomic displacement parameters (Å^2^)

**Table:**

	*U*^11^	*U*^22^	*U*^33^	*U*^12^	*U*^13^	*U*^23^
Zn1	0.0268 (2)	0.0244 (2)	0.0289 (2)	0.00223 (14)	0.00547 (14)	0.00410 (14)
O5	0.0308 (12)	0.0365 (13)	0.0356 (13)	0.0039 (10)	0.0020 (10)	0.0083 (10)
O3	0.0386 (13)	0.0318 (12)	0.0401 (13)	0.0035 (10)	0.0087 (11)	−0.0027 (10)
O4	0.0349 (12)	0.0303 (12)	0.0446 (13)	−0.0004 (10)	0.0136 (11)	0.0108 (10)
OW1	0.0350 (15)	0.0536 (16)	0.0338 (15)	0.0065 (12)	0.0050 (12)	0.0011 (12)
N2	0.0261 (13)	0.0256 (13)	0.0289 (14)	0.0040 (11)	0.0091 (11)	0.0031 (11)
N1	0.0236 (13)	0.0245 (13)	0.0328 (14)	0.0042 (10)	0.0061 (11)	0.0065 (11)
N3	0.0527 (18)	0.0371 (16)	0.0354 (16)	0.0053 (14)	0.0122 (14)	0.0025 (13)
C4	0.0263 (16)	0.0296 (16)	0.0292 (16)	0.0021 (13)	0.0052 (13)	0.0058 (13)
N4	0.0421 (17)	0.0427 (17)	0.0339 (16)	0.0041 (13)	0.0016 (13)	0.0101 (13)
C14	0.0228 (15)	0.0236 (15)	0.0274 (15)	0.0014 (12)	0.0060 (12)	0.0059 (12)
C8	0.0378 (18)	0.0279 (16)	0.0291 (17)	0.0018 (14)	0.0090 (14)	0.0025 (14)
C13	0.0260 (15)	0.0217 (15)	0.0292 (16)	0.0019 (12)	0.0072 (13)	0.0053 (13)
C12	0.0324 (17)	0.0325 (17)	0.0392 (18)	0.0091 (14)	0.0075 (14)	0.0119 (15)
C9	0.0323 (17)	0.0264 (16)	0.0324 (17)	0.0042 (13)	0.0108 (14)	0.0044 (13)
C5	0.0352 (18)	0.0326 (17)	0.0276 (16)	0.0006 (14)	0.0060 (14)	0.0078 (14)
C16	0.0361 (18)	0.0279 (16)	0.0365 (18)	0.0015 (14)	0.0083 (14)	0.0057 (14)
C19	0.0267 (16)	0.0268 (16)	0.0419 (19)	0.0029 (13)	0.0059 (14)	0.0084 (15)
C15	0.0403 (19)	0.0304 (18)	0.0350 (19)	0.0052 (15)	0.0127 (15)	0.0048 (15)
C10	0.0413 (19)	0.0342 (18)	0.0414 (19)	0.0124 (15)	0.0142 (16)	0.0013 (15)
C2	0.0313 (17)	0.0433 (19)	0.046 (2)	0.0154 (15)	0.0149 (15)	0.0145 (16)
C17	0.049 (2)	0.0263 (17)	0.0423 (19)	−0.0011 (15)	0.0166 (17)	0.0046 (15)
C3	0.0296 (17)	0.0410 (19)	0.0364 (18)	0.0103 (15)	0.0042 (14)	0.0142 (15)
C11	0.0358 (19)	0.0358 (19)	0.054 (2)	0.0137 (16)	0.0122 (17)	0.0087 (17)
C18	0.0419 (19)	0.0254 (17)	0.047 (2)	−0.0010 (15)	0.0146 (16)	0.0032 (15)
C1	0.0315 (17)	0.0321 (17)	0.0377 (18)	0.0086 (14)	0.0129 (14)	0.0045 (14)
C7	0.066 (3)	0.044 (2)	0.0270 (18)	0.0023 (19)	0.0057 (18)	−0.0015 (16)
C6	0.060 (2)	0.047 (2)	0.0292 (19)	−0.0039 (19)	−0.0007 (17)	0.0064 (17)
O2	0.0386 (13)	0.0284 (12)	0.0351 (12)	−0.0076 (10)	0.0089 (10)	0.0017 (10)
O1	0.087 (2)	0.0472 (16)	0.0611 (18)	−0.0163 (15)	0.0381 (17)	−0.0080 (14)

### Geometric parameters (Å, °)

**Table:**

Zn1—O2	1.987 (2)	C13—C9	1.394 (4)
Zn1—N1	2.120 (3)	C12—C11	1.390 (4)
Zn1—O5	2.137 (2)	C12—H12A	0.9300
Zn1—O4^i^	2.154 (2)	C9—C10	1.401 (4)
Zn1—N2	2.188 (2)	C16—C15	1.510 (4)
Zn1—O3^i^	2.347 (2)	C16—C17	1.513 (4)
Zn1—C19^i^	2.588 (3)	C16—H16A	0.9700
O5—H5A	0.8200	C16—H16B	0.9700
O5—H5B	1.00 (4)	C19—C18	1.514 (4)
O3—C19	1.237 (4)	C19—Zn1^ii^	2.588 (3)
O3—Zn1^ii^	2.347 (2)	C15—O1	1.223 (4)
O4—C19	1.276 (4)	C15—O2	1.272 (4)
O4—Zn1^ii^	2.154 (2)	C10—C11	1.363 (4)
OW1—HWBA	0.79 (4)	C10—H10A	0.9300
OW1—HWA1	0.85 (4)	C2—C3	1.358 (4)
N2—C1	1.330 (4)	C2—C1	1.400 (4)
N2—C14	1.345 (4)	C2—H2A	0.9300
N1—C12	1.329 (4)	C17—C18	1.518 (4)
N1—C13	1.351 (4)	C17—H17A	0.9700
N3—C7	1.314 (4)	C17—H17B	0.9700
N3—C8	1.367 (4)	C3—H3B	0.9300
C4—C14	1.401 (4)	C11—H11A	0.9300
C4—C3	1.401 (4)	C18—H18A	0.9700
C4—C5	1.459 (4)	C18—H18B	0.9700
N4—C6	1.314 (4)	C1—H1A	0.9300
N4—C5	1.352 (4)	C7—C6	1.401 (5)
C14—C13	1.463 (4)	C7—H7A	0.9300
C8—C5	1.401 (4)	C6—H6A	0.9300
C8—C9	1.452 (4)		
			
O2—Zn1—N1	114.24 (9)	C15—C16—C17	115.5 (3)
O2—Zn1—O5	92.64 (9)	C15—C16—H16A	108.4
N1—Zn1—O5	90.76 (9)	C17—C16—H16A	108.4
O2—Zn1—O4^i^	96.87 (9)	C15—C16—H16B	108.4
N1—Zn1—O4^i^	148.89 (9)	C17—C16—H16B	108.4
O5—Zn1—O4^i^	87.08 (8)	H16A—C16—H16B	107.5
O2—Zn1—N2	100.06 (10)	O3—C19—O4	120.8 (3)
N1—Zn1—N2	77.41 (9)	O3—C19—C18	121.2 (3)
O5—Zn1—N2	165.34 (9)	O4—C19—C18	118.0 (3)
O4^i^—Zn1—N2	98.53 (9)	O3—C19—Zn1^ii^	64.86 (16)
O2—Zn1—O3^i^	154.65 (8)	O4—C19—Zn1^ii^	56.11 (15)
N1—Zn1—O3^i^	91.04 (9)	C18—C19—Zn1^ii^	172.5 (2)
O5—Zn1—O3^i^	88.79 (9)	O1—C15—O2	122.9 (3)
O4^i^—Zn1—O3^i^	57.90 (8)	O1—C15—C16	120.1 (3)
N2—Zn1—O3^i^	82.87 (9)	O2—C15—C16	116.8 (3)
Zn1—O5—H5A	109.5	C11—C10—C9	120.1 (3)
Zn1—O5—H5B	111 (2)	C11—C10—H10A	120.0
H5A—O5—H5B	98.4	C9—C10—H10A	120.0
C19—O3—Zn1^ii^	86.64 (18)	C3—C2—C1	119.0 (3)
C19—O4—Zn1^ii^	94.44 (19)	C3—C2—H2A	120.5
HWBA—OW1—HWA1	96 (3)	C1—C2—H2A	120.5
C1—N2—C14	117.9 (3)	C16—C17—C18	113.3 (3)
C1—N2—Zn1	129.1 (2)	C16—C17—H17A	108.9
C14—N2—Zn1	112.93 (18)	C18—C17—H17A	108.9
C12—N1—C13	118.3 (3)	C16—C17—H17B	108.9
C12—N1—Zn1	126.4 (2)	C18—C17—H17B	108.9
C13—N1—Zn1	115.27 (18)	H17A—C17—H17B	107.7
C7—N3—C8	115.4 (3)	C2—C3—C4	119.8 (3)
C14—C4—C3	117.3 (3)	C2—C3—H3B	120.1
C14—C4—C5	120.3 (3)	C4—C3—H3B	120.1
C3—C4—C5	122.4 (3)	C10—C11—C12	118.6 (3)
C6—N4—C5	115.4 (3)	C10—C11—H11A	120.7
N2—C14—C4	123.1 (3)	C12—C11—H11A	120.7
N2—C14—C13	117.5 (2)	C19—C18—C17	114.4 (3)
C4—C14—C13	119.4 (3)	C19—C18—H18A	108.7
N3—C8—C5	121.1 (3)	C17—C18—H18A	108.7
N3—C8—C9	118.0 (3)	C19—C18—H18B	108.7
C5—C8—C9	120.8 (3)	C17—C18—H18B	108.7
N1—C13—C9	122.5 (3)	H18A—C18—H18B	107.6
N1—C13—C14	116.9 (2)	N2—C1—C2	122.9 (3)
C9—C13—C14	120.6 (3)	N2—C1—H1A	118.6
N1—C12—C11	123.0 (3)	C2—C1—H1A	118.6
N1—C12—H12A	118.5	N3—C7—C6	122.9 (3)
C11—C12—H12A	118.5	N3—C7—H7A	118.6
C13—C9—C10	117.5 (3)	C6—C7—H7A	118.6
C13—C9—C8	119.4 (3)	N4—C6—C7	122.9 (3)
C10—C9—C8	123.1 (3)	N4—C6—H6A	118.5
N4—C5—C8	122.2 (3)	C7—C6—H6A	118.5
N4—C5—C4	118.4 (3)	C15—O2—Zn1	119.5 (2)
C8—C5—C4	119.4 (3)		
			
O2—Zn1—N2—C1	−69.5 (3)	C14—C13—C9—C8	−1.3 (4)
N1—Zn1—N2—C1	177.7 (3)	N3—C8—C9—C13	−179.6 (3)
O5—Zn1—N2—C1	140.8 (3)	C5—C8—C9—C13	0.0 (4)
O4^i^—Zn1—N2—C1	29.1 (3)	N3—C8—C9—C10	−0.4 (5)
O3^i^—Zn1—N2—C1	85.0 (3)	C5—C8—C9—C10	179.2 (3)
C19^i^—Zn1—N2—C1	57.9 (3)	C6—N4—C5—C8	0.0 (5)
O2—Zn1—N2—C14	113.54 (19)	C6—N4—C5—C4	−179.4 (3)
N1—Zn1—N2—C14	0.73 (18)	N3—C8—C5—N4	0.5 (5)
O5—Zn1—N2—C14	−36.1 (4)	C9—C8—C5—N4	−179.1 (3)
O4^i^—Zn1—N2—C14	−147.87 (19)	N3—C8—C5—C4	179.9 (3)
O3^i^—Zn1—N2—C14	−91.94 (19)	C9—C8—C5—C4	0.2 (4)
C19^i^—Zn1—N2—C14	−119.0 (2)	C14—C4—C5—N4	−179.7 (3)
O2—Zn1—N1—C12	82.8 (3)	C3—C4—C5—N4	−0.4 (5)
O5—Zn1—N1—C12	−10.4 (2)	C14—C4—C5—C8	0.9 (4)
O4^i^—Zn1—N1—C12	−96.0 (3)	C3—C4—C5—C8	−179.8 (3)
N2—Zn1—N1—C12	178.3 (3)	Zn1^ii^—O3—C19—O4	4.3 (3)
O3^i^—Zn1—N1—C12	−99.2 (2)	Zn1^ii^—O3—C19—C18	−174.8 (3)
C19^i^—Zn1—N1—C12	−96.7 (3)	Zn1^ii^—O4—C19—O3	−4.7 (3)
O2—Zn1—N1—C13	−95.7 (2)	Zn1^ii^—O4—C19—C18	174.5 (2)
O5—Zn1—N1—C13	171.1 (2)	C17—C16—C15—O1	−13.0 (5)
O4^i^—Zn1—N1—C13	85.5 (3)	C17—C16—C15—O2	172.8 (3)
N2—Zn1—N1—C13	−0.17 (19)	C13—C9—C10—C11	−1.0 (5)
O3^i^—Zn1—N1—C13	82.3 (2)	C8—C9—C10—C11	179.8 (3)
C19^i^—Zn1—N1—C13	84.8 (2)	C15—C16—C17—C18	174.5 (3)
C1—N2—C14—C4	0.8 (4)	C1—C2—C3—C4	0.8 (5)
Zn1—N2—C14—C4	178.2 (2)	C14—C4—C3—C2	0.0 (5)
C1—N2—C14—C13	−178.5 (2)	C5—C4—C3—C2	−179.3 (3)
Zn1—N2—C14—C13	−1.2 (3)	C9—C10—C11—C12	1.3 (5)
C3—C4—C14—N2	−0.9 (4)	N1—C12—C11—C10	−0.8 (5)
C5—C4—C14—N2	178.4 (3)	O3—C19—C18—C17	8.1 (4)
C3—C4—C14—C13	178.4 (3)	O4—C19—C18—C17	−171.1 (3)
C5—C4—C14—C13	−2.2 (4)	Zn1^ii^—C19—C18—C17	−133.6 (16)
C7—N3—C8—C5	−0.4 (5)	C16—C17—C18—C19	175.0 (3)
C7—N3—C8—C9	179.2 (3)	C14—N2—C1—C2	0.1 (4)
C12—N1—C13—C9	0.2 (4)	Zn1—N2—C1—C2	−176.8 (2)
Zn1—N1—C13—C9	178.8 (2)	C3—C2—C1—N2	−0.9 (5)
C12—N1—C13—C14	−179.0 (2)	C8—N3—C7—C6	0.0 (5)
Zn1—N1—C13—C14	−0.4 (3)	C5—N4—C6—C7	−0.4 (5)
N2—C14—C13—N1	1.1 (4)	N3—C7—C6—N4	0.5 (6)
C4—C14—C13—N1	−178.3 (3)	O1—C15—O2—Zn1	1.6 (5)
N2—C14—C13—C9	−178.1 (3)	C16—C15—O2—Zn1	175.6 (2)
C4—C14—C13—C9	2.5 (4)	N1—Zn1—O2—C15	18.7 (3)
C13—N1—C12—C11	0.1 (4)	O5—Zn1—O2—C15	110.7 (2)
Zn1—N1—C12—C11	−178.3 (2)	O4^i^—Zn1—O2—C15	−161.9 (2)
N1—C13—C9—C10	0.2 (4)	N2—Zn1—O2—C15	−61.9 (2)
C14—C13—C9—C10	179.4 (3)	O3^i^—Zn1—O2—C15	−156.5 (2)
N1—C13—C9—C8	179.5 (3)	C19^i^—Zn1—O2—C15	−161.8 (2)

Symmetry codes: (i) *x*−1, *y*−1, *z*; (ii) *x*+1, *y*+1, *z*.

### Hydrogen-bond geometry (Å, °)

**Table:**

*D*—H···*A*	*D*—H	H···*A*	*D*···*A*	*D*—H···*A*
O5—H5A···OW1^iii^	0.82	1.89	2.702 (3)	173
O5—H5B···O4^iv^	1.00 (4)	1.91 (4)	2.856 (3)	157 (3)
OW1—HWA1···O4^v^	0.85 (4)	2.03 (4)	2.840 (4)	161 (3)
OW1—HWBA···O2	0.79 (4)	1.95 (4)	2.733 (4)	170 (4)

Symmetry codes: (iii) −*x*+1, −*y*, −*z*+1; (iv) −*x*+2, −*y*+1, −*z*+1; (v) −*x*+3, −*y*+1, −*z*+1.
